# Depth Density Achieves a Better Result for Semantic Segmentation with the Kinect System

**DOI:** 10.3390/s20030812

**Published:** 2020-02-03

**Authors:** Hanbing Deng, Tongyu Xu, Yuncheng Zhou, Teng Miao

**Affiliations:** 1 College of Information and Electrical Engineering, Shenyang Agricultural University, Shenyang 110866, China; denghanbing@syau.edu.cn (H.D.); zhouyc2002@syau.edu.cn (Y.Z.); miaoteng@syau.edu.cn (T.M.); 2 Liaoning Engineering Research Center for Information Technology in Agriculture, Shenyang 110866, China

**Keywords:** Kinect, depth density, fully convolutional networks, semantic segmentation

## Abstract

Image segmentation is one of the most important methods for animal phenome research. Since the advent of deep learning, many researchers have looked at multilayer convolutional neural networks to solve the problems of image segmentation. A network simplifies the task of image segmentation with automatic feature extraction. Many networks struggle to output accurate details when dealing with pixel-level segmentation. In this paper, we propose a new concept: Depth density. Based on a depth image, produced by a Kinect system, we design a new function to calculate the depth density value of each pixel and bring this value back to the result of semantic segmentation for improving the accuracy. In the experiment, we choose Simmental cattle as the target of image segmentation and fully convolutional networks (FCN) as the verification networks. We proved that depth density can improve four metrics of semantic segmentation (pixel accuracy, mean accuracy, mean intersection over union, and frequency weight intersection over union) by 2.9%, 0.3%, 11.4%, and 5.02%, respectively. The result shows that depth information produced by Kinect can improve the accuracy of the semantic segmentation of FCN. This provides a new way of analyzing the phenotype information of animals.

## 1. Introduction

In the field of bioscience, phenotype generally refers to the observable morphological characteristics of individuals or groups under specific conditions [1]. In the 1990s, with the development of gene research, products of gene expression, and various kinds of genetic association analyses, researchers proposed the concept of phenomics corresponding to genomics [2,3]. Since then, studies on single or serial phenomes of humans, animals, and plants have developed into an important branch of bioscience [4,5,6,7].

With the rapid development of computer vision technology, many researchers choose to use various visual devices to obtain animal and plant phenotypes, which can allow the machine to analyze their behavior and optimize the process of animal breeding and plant growing automatically [8,9]. At present, computer vision technology has been widely applied in animal and plant phenomics research. Improvements can be seen in many different aspects. Bauer used an automated and open-source analytic platform to combine modern computer vision, machine learning, and modular software engineering and measure yield-related phenotypes from ultra-large aerial imagery [10]. Mochida reviewed the emerging aspects of computer vision for automated plant phenotyping and give a machine learning perspective for improvement of plant productivity [11]. Prey evaluated RGB image and multispectral sensing for assessing early plant vigor [12]. Xiang created a non-destructive 3D scanning system to capture the sequential images of a plant at different heights [13]. Guan developed a low-cost, novel, and efficient imaging system for 3D reconstruction with color information [14]. Zhao proposed a high throughput prototype that combines stereo vision and grating dispersion to simultaneously acquire hyperspectral and 3D information [15]. However, traditional methods of computer vision interpret images by artificial features (color, texture, and shape in image) extractions [16,17]. If the content of an image is complex, it is very difficult to achieve artificial feature extraction, especially for image segmentation [18,19,20].

However, with the development of deep convolutional neural networks (DCNN), the concept (end-to-end) is introduced into computer vision [21,22]. Based on DCNN, computers learn and find the most descriptive and prominent features in each image automatically [23,24,25]. The structure of neural networks can discover and remember the potential patterns of various objects in an image. Based on big data, DCNN can be trained sufficiently to give a high accuracy output. With this model, the main cost is transferred from algorithm design to data collection [26]. Therefore, for the research of animal phenomes, DCNN has become the main choice in computer vision technology. Hu proposes a deep learning-based method to gain an accurate count of wheat ears and spikelets. He improves the generator‘s learning ability and prediction accuracy for occluded wheat ears [27]. Lee used image processing and machine learning to distinguish ten Fagaceae species. The results indicated that the proposed approach had an accuracy of 92.8% [28]. Jin proposed a computerized system that is capable of detecting Fusarium wilt of radishes with high accuracy [29]. Andres proposed a system that combines vegetation detection and deep learning to obtain a high-quality classification of the vegetation in the field into value crops and weeds [30].

Object recognition and detection are two areas of focus in computer vision. However, based on DCNN, most object recognition and detection methods are implemented by bounding box methods, such as RCNN series networks [31,32,33], SPPNet [34], YOLO [35], and many other detection deep learning models [36], which are quite different from human vision [37]. With small perception regions in shallow layers, DCNN can only learn partial features in images. As the convolutional layers go deeper, more abstract features can be obtained by the larger perception regions. These abstract features are less sensitive to size, location, and orientations of the object. Neural networks can realize classification more easily with these features [38]. 

The methods of image segmentation based on DCNN usually classify a pixel with an around kernel region as the input for training and prediction. However, there are several disadvantages to these methods. The cost of storage is large. An *n* × *n* size kernel region for each pixel needs an additional *n*^2^ storage space. The efficiency of computation is low. During the training, for each pixel, the adjacent kernel regions have large overlapping areas, which leads to repeated computation. The size of the kernel region limits the size of the perception area. Usually, the size of the kernel region is much smaller than the whole image. Therefore, limited partial features can be extracted, and this leads to the limitation of the classification performance. In addition, DCNN loses some details during the training process. It cannot point out which object the pixels belong to. For this reason, it is difficult for DCNN to implement the classification task at the pixel level. 

To avoid these shortcomings, Evan proposed a fully convolutional network (FCN) to realize image segmentation at the pixel level (semantic segmentation). The FCN attempts to recover the category of each pixel from the abstract feature maps, which transforms the classification task from the image level to the pixel level [39]. Compared with other segmentation methods of DCNN, FCN can accept input images of arbitrary size, without requiring all the training and testing samples to have the same scale. FCN avoids the problem of repeated computation and storage waste for prediction.

However, the shortcomings of FCN are also obvious: The accuracy of semantic segmentation is poor. The results generated by up-sampling are still fuzzy and insensitive to details. The FCN does not take full account of the relationship between pixels. It neglects the spatial regularization and lacks spatial consistency. Because FCN does not record the position relationship of pixels before and after the forward convolution. In the process of the up-sampling of FCN, the effective pixels of the heat maps will be restored to the random position in the segmentation results.

To solve these problems, we propose a new concept: Depth density. Based on the depth image, produced by a Kinect system, a new function is designed to calculate the depth density value of each pixel in depth images. We use the value of depth density to define the probability that a pixel belongs to the object or the boundary of the object. This method can partially solve the problem of fuzzy boundaries and detail insensitivity for FCN. The experiment shows that the four metrics of semantic segmentation have obvious increments.

## 2. Methods

### 2.1. Experimental Materials and Setup

We chose Simmental cattle as the target of semantic segmentation. In order to increase the variety of images, the experiment was carried out in May, August, and October in Shenyang, China. The images of cattle were obtained from the indoor and outdoor environment during three periods (8:00–10:00, 11:00–13:00, and 14:00–16:00). 

We selected the Kinect sensor (V2.0) to acquire the RGB (three-primary colors) image and depth image simultaneously. With TOF (time of flight), Kinect can calculate the distance between the object and camera and give a value for each pixel in depth images. However, Kinect is easily disturbed by luminous beams, which create lots of noise when using this device in high light intensity environments. 

For this experiment, in order to reduce the influence of noise on the depth images, we choose to carry out the image collection in a light controllable environment (indoor scene without direct sunlight). There were 30 cows as the candidates in this experiment. We equally divided them into several groups. During the process of data acquisition, the Kinect is located at a fixed distance. The subjects (cattle) kept a distance of 0.5–4.5 m from the camera. In the same time trajectory, RGB images and depth images were extracted from a Kinect video stream at equal intervals (five frames per second). We labeled the RGB images and divided them into training and testing samples. We selected 3000 images for training and 600 images for testing.

### 2.2. Fully Convolutional Networks (FCN) and up-Sampling

**Figure 1:**
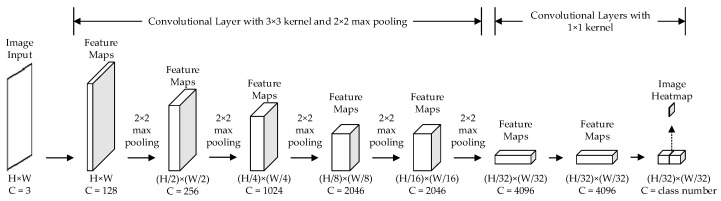
The procedure for creating heat maps based on FCN. *H* indicates the height of feature maps, *W* indicates the width of feature maps, and *C* indicates the channel number of the convolutional kernel.

The classification network is the basic model for semantic segmentation. Therefore, we selected VGG-19 [40] as the basic network, which was born in ILSVRC-2014 [41]. The structure of VGG-19 proved that the number of layers is the key to realize excellent performance for DCNN. However, during the training of VGG-19, lots of pixel information was dropped, and the model could not achieve prediction at the pixel level. To solve these problems, FCN converts the last three fully connected layers into convolutional layers. The kernel size of fully convolutional layers is set into 1 × 1, and the number of channels remains unchanged. The convolutional layer can retain the spatial property of feature maps and output the classification results, which are called heat maps. Different classes are represented with different gray values, which can be shown in heat maps. Through max-pooling (size = 2 × 2), the heat map would be resized into 1/32 of the input’s size. The details of FCN are shown in Figure 1.

**Figure 2:**
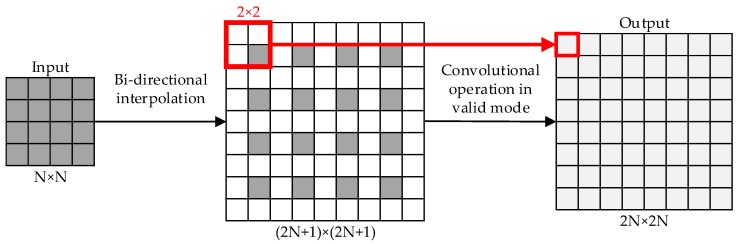
Bilinear with the valid mode (N = 4 in this example). The size of the input feature map is *N* × *N*. We expand the original size of the feature map to (2*N* + 1) × (2*N* + 1) and set the value of intervals with 0. Then, we use a 2 × 2 kernel to carry out the convolutional operation in the “valid” mode (padding = 0 and step = 1), which could obtain a new 2*N* × 2*N* output.

**Figure 3:**
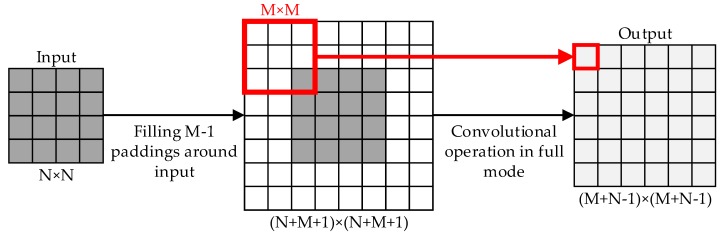
De-convolution with the full mode (N = 4, M = 3 in this example). The size of the input feature map is *N* × *N*. We expand the original feature maps with *M* − 1 paddings (padding pixel = 0) around. Then, we use an *M* × *M* kernel to carry out the convolutional operation with the “full” mode (padding = *M* − 1 and step = 1), which could obtain an (*M* + *N* − 1) × (*M* + *N* − 1) output.

The heat maps cannot be directly used in semantic segmentation. The size of the heat maps has to be expanded to the same size as the input images. We used up-sampling to finish that process. Up-sampling can be seen as the inverse process of pooling and can increase the map’s data quantity. There are three basic up-sampling operations in computer vision: Bilinear [42], de-convolution [43], and de-pooling [44]. In this paper, unlike FCN, we alternately use bilinear and de-convolution during the up-sampling, and use the “valid” mode in bilinear and the “full” mode in de-convolution. Figure 2 shows the details of bilinear and Figure 3 shows the details of de-convolution.

**Figure 4:**
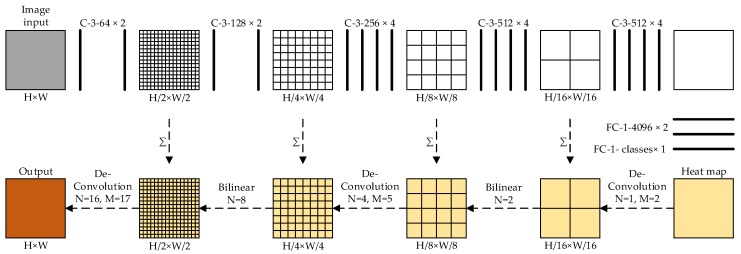
Up-sampling with bilinear and de-convolution operations. *H* indicates the height of the feature maps, *W* indicates the width of the feature maps. *N* indicates the pixel side length of the feature maps. *M* indicates the size of the convolutional kernel in de-convolution.

There are five max-pooling layers in FCN. The size of the feature map will reduce to a quarter of the original size after passing one max-pooling layer. In Figure 4, the size of the input is *H* × *W*. By five max-pooling operations, the size of the heat map becomes *H*/32 × *W*/32. In order to illustrate the steps of up-sampling, we assume that the size of the input is 32 × 32. First, we handle the heat map with de-convolution operations (*N* = 1, *M* = 2) and expand its size from 1 × 1 to 2 × 2. Then, we handle the intermediate map with the bilinear operation to expand its size from 2 × 2 to 4 × 4. To enrich the content of the heat map, it would be better to add a heat map and a feature map, which is generated during forward convolutional operations before each operation. We use these two operations alternately until the size of the heat map is equal to the size of the original input. Finally, each value of gray is assigned a unique category so that the whole image achieves segmentation at the pixel level.

### 2.3. Depth Density

The up-sampling of FCN has two problems, pixel misclassification, and pixel loss. Therefore, we attempt to use the depth channel of RGBD (RGB and Depth) images (from the Kinect) to solve these problems.

A depth image is a pseudo image whose depth of pixels are converted from the distance between object and camera. We transform the depth value into the normalized gray value. In a depth image, the range of depth is [0, 1]. Pixels that come from the same object would have a continuous gradient of depth. Conversely, pixels that come from the boundary between the adjacent objects would have a large gradient change.

Therefore, in order to establish the relationship between adjacent discrete pixels, we propose a new concept: Depth density. The value of depth density can represent the probability that a pixel belongs to the category of its adjacent pixel region.

We set ***D*** to indicate the depth image with the size of *h* *× w*. *h* indicates the row’s number of ***D***, and *w* indicates the column’s number of ***D***. (*r*, *c*) indicates the position index of a pixel in ***D***. *r* denotes the row coordinates and *c* denotes the column coordinates of ***D***. *d_r,c_* indicates the depth value of a pixel (*r*, *c*) in ***D***. *dd_r,c_* indicates the value of the depth density for a pixel (*r*, *c*) in ***D***. $K_{r,c}^{s}$ indicates the partial region of pixels in ***D***. Its central coordinate is (*r*, *c*) and its area is *s*. The equation of depth density ($dd_{r,c}$) is shown in Equation (1):

(1) $$dd_{r,c}=f\left(d_{r,c},K_{r,c}^{s}\right).$$

Equation (1) shows that there are two parameters ($d_{r,c}\;\mathrm{and}\;K_{x,y}^{s}$) that could affect depth density. For $K_{r,c}^{s}$, there are two factors that need to be considered:

- The average depth of $K_{r,c}^{s}$, and
- The depth distribution of the central pixel and its adjacent pixels in $K_{r,c}^{s}$.

In order to obtain the solution of depth density, we set ${\bar{d}}_{r,c}$ as the average depth of $K_{r,c}^{s}$ and the equation is shown in Equation (2):

(2) $${\bar{d}}_{r,c}=\sum_{i}\sum_{j}d_{i,j}/s,$$

the range of *i* is $\left[r-\sqrt{s},r+\sqrt{s}\right]$ and the range of *j* is $\left[c-\sqrt{s},c+\sqrt{s}\right]$. Then we set ${\bar{\sigma }}_{r,c}$ as the standard deviation of depth in $K_{r,c}^{s}$ and ${\sigma^{\prime }}_{r,c}$ as the central deviation of depth in $K_{r,c}^{s}$. The two equations are shown in Equations (3) and (4) separately:

(3) $${\bar{\sigma }}_{r,c}=\sqrt[{2}]{\frac{\sum_{i}\sum_{j}{\left(d_{i,j}-{\bar{d}}_{r,c}\right)}^{2}}{s}},$$

(4) $${\sigma^{\prime }}_{r,c}=\sqrt[{2}]{\frac{\sum_{i}\sum_{j}{\left(d_{i,j}-d_{r,c}\right)}^{2}}{s}}.$$

We fill the surrounding region of images with $\left(\sqrt{s}-1\right)/2$ paddings (gray value = 0) to handle the pixels on the sides of ***D***. The row’s number of ***D*** will change to $h+\left(\sqrt{s}-1\right)/2$ and the column’s number of ***D*** will change to $w+\left(\sqrt{s}-1\right)/2$. With ${\bar{d}}_{r,c}$, ${\bar{\sigma }}_{r,c}$ and ${\sigma^{\prime }}_{r,c}$, we can get the depth density of every pixel using Equation (5):

(5) $$dd_{r,c}=\frac{Gs\left(d_{i,j},{\bar{d}}_{r,c},{\bar{\sigma }}_{r,c}\right)\times Gs\left(d_{i,j},d_{r,c},{\sigma^{\prime }}_{r,c}\right)}{{\left(\underset{}{\max}\left[Gs\left({\bar{d}}_{r,c},{\bar{d}}_{r,c},{\bar{\sigma }}_{r,c}\right),Gs\left(d_{r,c},d_{r,c},{\sigma^{\prime }}_{r,c}\right)\right]\right)}^{2}},$$

where the range of *i* is $\left[r-\sqrt{s},r+\sqrt{s}\right]$, the range of *j* is $\left[c-\sqrt{s},c+\sqrt{s}\right]$. Gs(x,μ,σ) is a standard Gauss function, which is shown in Equation (6):

(6) $$Gs\left(x,\mu ,\sigma \right)=\frac{1}{\sigma \sqrt{2\pi }}e^{-\frac{{\left(x-\mu \right)}^{2}}{2\sigma^{2}}}.$$

$Gs\left(d_{i,j},{\bar{d}}_{r,c},{\bar{\sigma }}_{r,c}\right)$ can be regarded as the key factor to measure the difference between $d_{r,c}$ and ${\bar{d}}_{r,c}$ of $K_{r,c}^{s}$. $Gs\left(d_{i,j},d_{r,c},{\sigma^{\prime }}_{r,c}\right)$ can be regarded as the key factor to measure the difference between $d_{r,c}$ and other surrounding pixels of $K_{r,c}^{s}$. 

### 2.4. Analysis and Improvement of Depth Density

**Figure 5:**
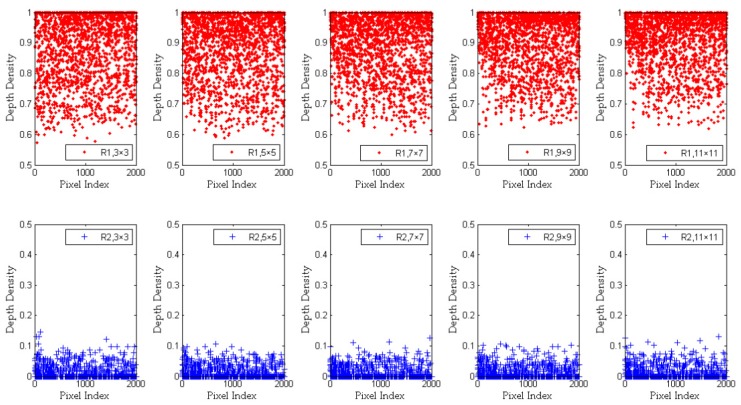
The depth density of all pixels in R1 and R2 using Equation (5). We set the size *s* to 3 × 3, 5 × 5, 7 × 7, 9 × 9, and 11 × 11, respectively. The depth density range of R1 is [0, 1]. The depth density range of R2 is [0, 0.5]. The pixel index represents the total number of pixels obtained by R1 or R2.

According to Equation (1), we select several group parameters to calculate the value of depth density and set the size *s* to 3 × 3, 5 × 5, 7 × 7, 9 × 9, and 11 × 11, respectively. Then, we crop two regions (R1 and R2) from the same original depth image. R1 (which comes from the surface of an object) contains a continuous gradient of depth. R2 (which comes from the boundary between two objects) contains a large gradient change. Figure 5 shows the depth density distribution of R1 and R2 with different kernel sizes.

In the region of R1, all pixels come from the surface of the same object. So we know that the adjacent pixels have similar values of depth. Therefore, when calculating $dd_{r,c}$, the values of $Gs\left(d_{i,j},{\bar{d}}_{r,c},{\bar{\sigma }}_{r,c}\right)$, $Gs\left({\bar{d}}_{r,c},{\bar{d}}_{r,c},{\bar{\sigma }}_{r,c}\right)$, $Gs\left(d_{i,j},d_{r,c},{\sigma^{\prime }}_{r,c}\right)$ and $Gs\left(d_{r,c},d_{r,c},{\sigma^{\prime }}_{r,c}\right)$ are close. So as shown from the top 5 graphs of Figure 5, the depth density value of R1 is distributed in the range [0.6, 1]. On the contrary, in the region of R2, there are a large number of boundary pixels that contain big gaps in depth value. The values of $Gs\left(d_{i,j},{\bar{d}}_{r,c},{\bar{\sigma }}_{r,c}\right)$, $Gs\left({\bar{d}}_{r,c},{\bar{d}}_{r,c},{\bar{\sigma }}_{r,c}\right)$, $Gs\left(d_{i,j},d_{r,c},{\sigma^{\prime }}_{r,c}\right)$, and $Gs\left(d_{r,c},d_{r,c},{\sigma^{\prime }}_{r,c}\right)$ are quite different. As shown from the bottom five graphs of Figure 5, the depth density value of R2 is distributed in the range [0, 0.15]. 

Since the influence of pixel space distance on the depth density is not considered in Equation (5), $dd_{r,c}$ does not change much with the increase of *s*. Therefore, we propose a new concept of “pixel distance difference” in this paper, where the value can be shown by Equations (7) and (8):

(7) $${\bar{dif}}_{i,j}=\frac{\left|d_{i,j}-{\bar{d}}_{r,c}\right|}{dist_{i.j}},$$

(8) $$di{f^{\prime }}_{i,j}=\frac{\left|d_{i,j}-d_{r,c}\right|}{dist_{i,j}},$$

in Equations (7) and (8), $dist_{i,j}$ indicates the distance between pixel (*i*, *j*) and pixel (*r*, *c*), and the value of $dist_{i,j}$ is shown in Equation (9):

(9) $$dist_{i,j}={\left|r-i\right|}_{i\in K_{r,c}^{s}}+{\left|c-j\right|}_{j\in K_{r,c}^{s}}.$$

We import Equations (7) and (8) into Equation (5), and get a new improvement of $dd_{r,c}$, which is shown in Equation (10):

(10) $$dd_{r,c}=\frac{Gs\left(d_{i,j}-{\bar{dif}}_{i,j},{\bar{d}}_{r,c},{\bar{\sigma }}_{r,c}\right)\times Gs\left(d_{i,j}-di{f^{\prime }}_{i,j},d_{r,c},{\sigma^{\prime }}_{r,c}\right)}{{\left(\underset{}{\max}\left[Gs\left({\bar{d}}_{r,c},{\bar{d}}_{r,c},{\bar{\sigma }}_{r,c}\right),Gs\left(d_{r,c},d_{r,c},{\sigma^{\prime }}_{r,c}\right)\right]\right)}^{2}}.$$

**Figure 6:**
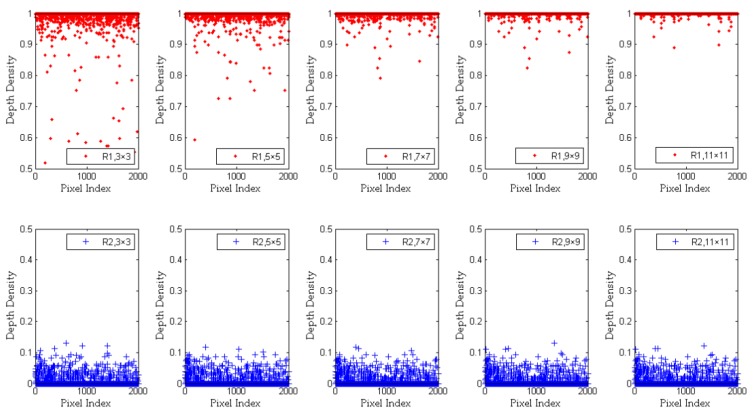
The depth density of all pixels in R1 and R2 by Equation (14). We set the size *s* to 3 × 3, 5 × 5, 7 × 7, 9 × 9, and 11 × 11, respectively. The depth density range of R1 is [0, 1]. The depth density range of R2 is [0, 0.5]. The pixel index represents the total number of pixels obtained by R1 or R2.

We use Equation (14) to recalculate the depth density of R1 and R2. For R1, as shown at the top five graphs of Figure 6, the distance factor is taken into account in Equation (14). The depth difference between pixels with a long distance is reduced by ${\bar{dif}}_{i,j}$ and $di{f^{\prime }}_{i,j}$ when *s* is increasing. This also reduces the influence of noise when calculating $dd_{r,c}$ with a smaller *s*. However, there are still some fluctuations, which are mainly caused by the noise in this region. With the increase of s, most noises regarding image depth are removed. Thus, besides the noise, the depth density values of pixels from the same object surface are distributed between 0.9 and 1. For R2, in the depth image, there are lots of zero-value pixels near the boundary between different objects. Therefore, the depth density values in this region are closer to 0. It has been calculated that most pixels of R2 have the depth density value range of [0, 0.15].

The higher the depth density value of a pixel, the higher the probability that it belongs to the same category as other surrounding pixels. On the contrary, the lower the depth density value of a pixel, the higher the probability that it belongs to different objects. In addition, the size of the $K_{r,c}^{s}$ also affects $dd_{r,c}$. When the size is small, the method is more sensitive to the edge or tiny pixels of an object. When the size is large, the method is more sensitive to the consecutive surface of the object. Depth density can prove that the spatially adjacent pixels have an approximate value of depth density and likely belong to the same object in the image. Based on this principle, we can use depth density to refine the results of semantic segmentation with FCN.

## 3. Results

### 3.1. Training of FCN

**Figure 7:**
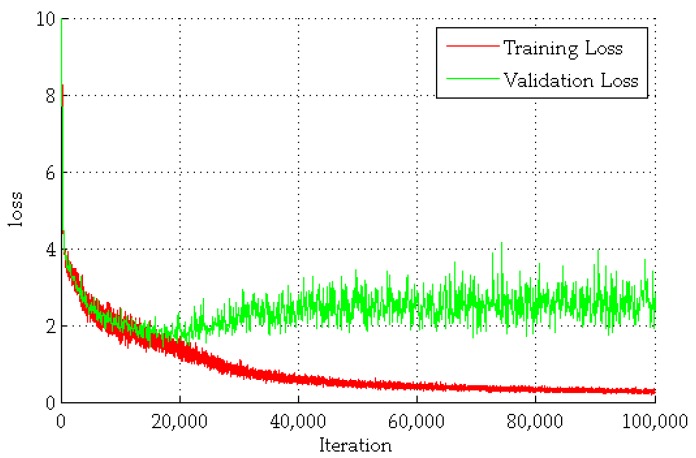
The losses of training and validation with FCN (fully convolutional networks). The abscissa represents the number of training batches. The ordinate represents the value of training or validation loss.

The training of FCN is carried out by optimizing the multinomial logistic regression objective using a mini-batch gradient descent with momentum. The batch size is set to 64. Momentum is set to 0.9. The learning rate is initially set to 0.001, and then decreased by a factor of 10 when the accuracy of the validation set stops improving. The learning rate could decrease three times, and learning would stop after 100,000 iterations. Figure 7 shows the training loss and validation loss of FCN (based on VGG-19). Since the scale of the data set used in this experiment is far less than in ImageNet, the loss has essentially reached the classification requirement after 70,000 iterations.

### 3.2. Metrics of Semantic Segmentation

We report four metrics from the common semantic segmentation and scene parsing evaluations that are variations on pixel accuracy and the region intersection over union (IoU). All the metrics are shown in Equations (11)–(14):

(11) $$\mathrm{pa}=\frac{\sum_{i}n_{ii}}{\sum_{i}t_{i}},$$

(12) $$\mathrm{ma}=\frac{1}{n_{cl}}\times \underset{i}{\sum }\frac{n_{ii}}{t_{i}},$$

(13) $$\mathrm{mIU}=\frac{1}{n_{cl}}\times \sum_{i}\frac{n_{ii}}{t_{i}+\sum_{j}n_{ji}-n_{ii}},$$

(14) $$f.w.\mathrm{IU}=\frac{1}{\sum_{k}t_{k}}\times \sum_{i}\frac{t_{i}\times n_{ii}}{t_{i}+\sum_{j}n_{ji}-n_{ii}},$$

where $n_{ij}$ is the number of pixels of class-i predicted to belong to class-j. $t_{i}=\underset{j}{\sum }n_{ij}$ indicates the total number of pixels of class-I, and $n_{cl}$ is the total number of class. We use ‘pa’, ‘ma’, ‘mIU’, and ‘f.w.IU’ to be the abbreviations of ‘pixel accuracy’, ‘mean accuracy’, ‘mean intersection over union ’, and ‘frequency weighted intersection over union’.

### 3.3. The Improvement of Semantic Segmentation by Depth Density

In order to get a better semantic segmentation result, we want to use the depth density of depth images to improve the output accuracy of up-sampling with FCN. In Section 2.4, we already proved that if the depth density of a pixel is high, there is a high probability that it could be the same category as its surrounding pixels. Therefore, we refine our segmentation results based on this principle.

**Figure 8:**
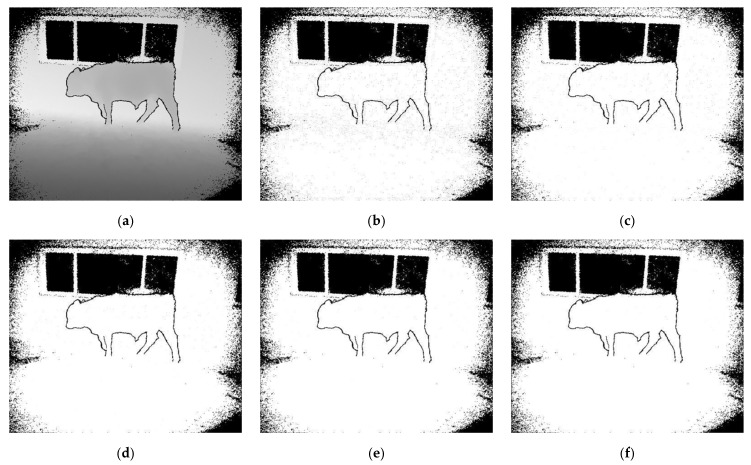
Pseudo images of depth image processed by the depth density algorithm. We set the size s to 3, 5, 7, 9, and 11, respectively. The value range of the depth density range is [0, 1]. (**a**) The original depth image. (**b**) The pseudo image of depth density (s = 3). (**c**) The pseudo image of depth density (s = 5). (**d**) The pseudo image of depth density (s = 7). (**e**) The pseudo image of depth density (s = 9). (**f**) The pseudo image of depth density (s = 11).

According to Equation (10), in order to have a moderate computation cost of this algorithm without accuracy loss, we set the s value to 3, 5, 7, 9, and 11, respectively, and use the depth density value of each pixel to generate the pseudo image. As shown in Figure 8, we compare a series of test results and the running time of the program. With the increase of s, the noises on the surface of the object decrease (the body area of cattle in image), while the boundary of the object is still clear. However, the increase of s adds to the running time of the program (as shown in Table 1). Considering the effect and time cost of this algorithm, we set s = 7.

FCN-8s (the best result of FCN) was selected to verify the effectiveness of this algorithm. We used the depth density of pixels to adjust the category of pixels. If the value of depth density were in the range of [0.9, 1], the pixel would have the same category as the surrounding pixels with a high probability. If the value of depth density were in the range of [0, 0.15], the pixel’s category would belong to the boundary, noise, or background with a high probability. We set all these kinds of pixels to the category of background. With depth density, the algorithm can remove discrete noise pixels of the object surface when $dd_{r,c}$ is less than 0.9 and remove noise pixels around the boundary between objects when $dd_{r,c}$ is less than 0.2. 

**Figure 9:**
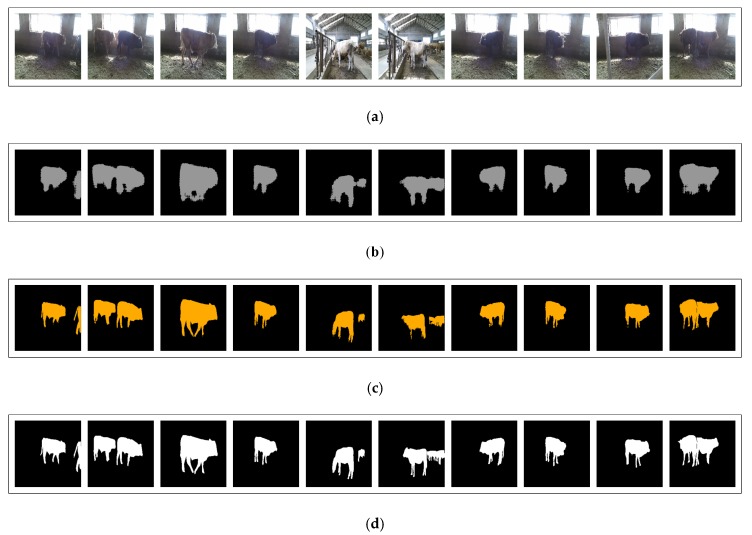
The comparison of semantic segmentation results. (**a**) Original RGB images. (**b**) The semantic segmentation results of fully convolutional networks. (**c**) The semantic segmentation results of depth density. (**d**) Ground truth of semantic segmentation.

As shown in Figure 9, compared with FCN-8s, the algorithm gives a better result for semantic segmentation, which is closer to the ground-truth. After the processing by depth density, the appearance of boundary blur in the original FCN-8s is distinctly improved. This is mainly due to the fact that the pixels with small spatial distance can be re-clustered by depth density. We judge the correlation between two pixels according to the depth values and the spatial distance on the image, and give the probability that two pixels belong to the same category. Using this probability, the results of FCN semantic segmentation can be improved.

With the validation dataset, we calculated the four metrics of semantic segmentation results. As shown in Table 2, the method improved on four metrics with depth density. Pixel accuracy increased by 2.9%. Mean accuracy increased by 0.3%. The mean intersection over union increased by 11.4%. The frequency weight intersection over union increased by 5.02%. This indicates that the depth density can be used to optimize the semantic segmentation results of the FCN and improve the segmentation accuracy.

## 4. Discussion

In this paper, we use depth images to prove that the depth information of images can be used to improve the effect of semantic segmentation with fully convolutional networks. The principle is to use the depth change between two pixels in the depth image to determine whether these two pixels come from the same object. Therefore, the concept of depth density is proposed in this paper. Using the depth value distribution of surrounding pixels, we can calculate the depth density of each pixel in the depth image. The value of depth density can directly reflect whether the pixel has the same category of the other pixels in this region. In the process of designing the depth density function, we add a concept of “pixel distance difference”, which increases the threshold value of judging whether a pixel belongs to the same category from [0.6, 1] to [0.9, 1]. This improves the accuracy of pixel classification.

When comparing the effect of segmentation between the method of depth density and FCN, four metrics are selected, respectively: Pixel accuracy (pa), mean accuracy (ma), mean intersection over union (mIU), and frequency weight intersection over union (f.w.IU). We found that the depth density of pixels can be used to re-classify the misclassified pixels in the semantic segmentation results of FCN, and the four metrics are increased by 2.9%, 0.3%, 11.4%, and 5.02%, respectively. Among these four metrics, mIU increases the most. As the pixels belonging to the background and the ones belonging to objects (cattle in this paper) can be clearly separated using the value of depth density, many misclassified pixels are corrected. However, the improvement of ma is relatively small. As we test the effect of semantic segmentation based on one category (cattle) in this paper, there are only two types of pixels in any single image. Therefore, in the calculation of ma, the category of pixels can only belong to the background or cattle, so the improvement of ma is small. This problem will be significantly solved after an increase in category number.

We analyze the image of semantic segmentation and find that the output of FCN can only give the basic shape of cattle, such as the position of head, legs, and trunk. The details of cattle are not clear. When we use the depth density of the pixel to refine the output of FCN, it can be seen that the details of cattle are clearer than before, especially the pixels of limbs. From the perspective of human vision, this result is very close to the ground truth. We provide a new method to improve the accuracy of semantic segmentation of fully convolutional networks and prove that using multimodal methods (for example: Depth and RGB in this paper) can achieve a better result with deep convolutional neural networks.

## 5. Conclusions

In this paper, we propose a new method for solving a problem (blur in semantic segmentation) of FCN. We alternately use bilinear (valid mode) and deconvolution (full mode) during up-sampling to get better results than using deconvolution only. In order to avoid the misclassification of pixels and increase the accuracy of FCN, we created an algorithm to calculate the depth density of pixels based on the depth images. The value of depth density can be used to determine whether pixels belong to the objects or background. This method can remove most noise pixels from the surface of an object. Compared with the results of FCN, this method is more sensitive to the boundary and can obtain a clearer visualization effect. The experimental results show that the pixel accuracy is improved by 2.9%, the mean accuracy is increased by 0.3%, the mean intersection over union is increased by 11.4%, and the frequency weight intersection over union is increased by 5.02%. These results prove that using depth density can improve the segmentation accuracy of FCN.

## Figures and Tables

**Table 1:** The time cost for the algorithm to process one image, when s takes different values.

The Value of s	Time Cost (s)
s = 3	16
s = 5	37
s = 7	69
s = 9	111
s = 11	163

**Table 2:** Four metrics of FCN-8s and the refined results by depth density on the validation set.

Results of Method	pa ^1^	ma ^2^	mIU ^3^	f.w.IU ^4^
FCN-8s	0.963	0.961	0.857	0.935
Depth density	0.991	0.964	0.955	0.982
Increment	2.9%	0.3%	11.4%	5.02%

^1^ pa: Pixel accuracy. ^2^ ma: Mean accuracy. ^3^ mIU: Mean intersection over union. ^4^ f.w.IU: Frequency weight intersection over union.
